# Supplementary material for: Frailty and loneliness among community-dwelling older adults: examining reciprocal associations within a measurement burst design
Source: BMC Geriatr. 2025 Mar 1;25:139. doi: 10.1186/s12877-025-05808-w (PMC11872317; doi:10.1186/s12877-025-05808-w)
Supplement: Supplementary file 1 — Supplementary Material 1 [file 12877_2025_5808_MOESM1_ESM.pdf]

Supplementary Material

Contents

Supplementary Methods 1: Data collection process

Supplementary Figure 1: Interview schedule

Supplementary Table 1: Health deficits considered in the FI

Supplementary Table 2: UCLA loneliness scale

Supplementary Methods 2: Model selection

Supplementary Figure 2: Correlation between FI and LS

Supplementary Table 3: Univariate FI models

Supplementary Table 4: Comparison of FI models

Supplementary Table 5: Univariate LS models

Supplementary Table 6: Comparison of LS models

Supplementary Table 7: Multiple group analysis with regard to sex

Supplementary Table 8: Multiple group analysis with regard to age

Supplementary Table 9: Multiple group analysis with regard to living alone

Supplementary Table 10: Multiple group analysis with regard to social participation

Supplementary Table 11: LCM-SR with bedrest as time varying covariate

Supplementary Table 12: LCM-SR with falls as time varying covariate

Supplementary References

Supplementary Methods 1: Data collection process

A professional survey agency contacted community-dwelling older adults based on previous participation in population representative surveys. Interviewers explained the study's topic, duration (i. e., two rounds of seven biweekly interviews spaced one year apart), and the information required, ensured the anonymity of all personal data, and obtained written consent from participants before participation.

The first interview of the first burst lasted a median of 23.9 minutes and was conducted in person (computer-assisted personal interview; CAPI) at the homes of the older adults by the end of August 2021. The subsequent computer assisted telephone interviews (CATI; waves 2 to 7) lasted between 8.7 and 9.5 minutes (median; Stolz, 2024). With a total of 40 participants, all interviews were conducted in person due to continued physical performance tests (i. e., grip strength, gait speed, and chair rise; physical performance tests are not considered in the current analysis). The mean duration of the first burst was 87 days (SD=13), i. e., on average, the interviews were repeated at intervals of 14.5 days.

Interviews of the second burst (w8–w14) stared in November 2022 (i. e., one year after the end of the first burst; the average number of days between w7 and w8 was 376, SD=11). Again, the first interview of the second burst (w8) was conducted in person (CAPI) at the home of the older adult and lasted a median of 15.1 minutes. The subsequent interviews (w9–w14) were conducted via telephone (CATI) and lasted between 7.9 and 8.7 minutes (median; Stolz, 2024). The mean duration of the second burst was 76 days (SD=11), i. e., on average, the interviews were repeated at intervals of 12.7 days.

Supplementary Figure 1: Interview schedule in which individuals and completed interviews are depicted with gray lines and black circles, respectively

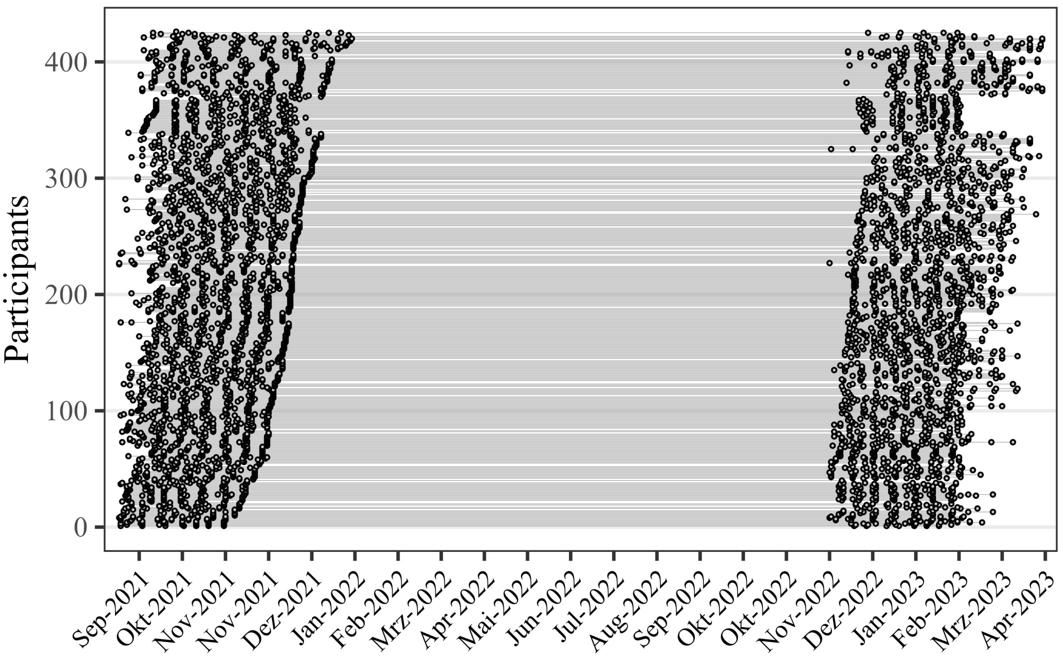

Supplementary Table 1: Health deficits considered in the frailty index

	health deficit	assigned values	prevalence at baseline (%)	missing data at baseline (%)
1	Self-rated health	0 = excellent<br>0.25 = very good<br>0.5 = good<br>0.75 = moderate<br>1 = poor	0 = 6.3<br>0.25 = 18.5<br>0.5 = 35.9<br>0.75 = 28.4<br>1 = 10.8	–
2	Dizziness	0 = no/1 = yes	1 = 20.7	–
3	Pain (rating from 0–10)	0 = 0<br>0.5 = ≥ 1 & ≤3<br>1 = ≥	0 = 26.1<br>0.5 = 35.2<br>1 = 38.7	–
4	Tiredness	0 = never<br>0.5 = sometimes<br>1 = often	0 = 43.0<br>0.5 = 42.0<br>1 = 15.0	–
5	Vision	0 = excellent<br>0.25 = very good<br>0.5 = good<br>0.75 = moderate<br>1 = poor	0 = 10.4<br>0.25 = 38.0<br>0.5 = 35.1<br>0.75 = 13.4<br>1 = 3.1	0.5
6	Hearing	0 = excellent<br>0.25 = very good<br>0.5 = good<br>0.75 = moderate<br>1 = poor	0 = 12.7<br>0.25 = 36.2<br>0.5 = 30.8<br>0.75 = 16.9<br>1 = 3.3	0.2
7	Attention (10 words immediate recall test)	0 = ≥ 5 words<br>1 = < 5 words	1 = 20.0	–
8	Memory (10 words delayed recall test)	0 = ≥ 4 words<br>1 = < 4 words	1 = 32.6	–
9	Physical inactivity (moderate physical activity)	0 = every day/almost every day & multiple times a week<br>1 = once per week & less often	1 = 21.4	–
Doctor told you had: . . .				
10	Heart problem (myocardial infarction, coronary thrombosis, other problem including congestive heart failure)	0 = no/1 = yes	1 = 15.3	–
11	High blood pressure or hypertension	0 = no/1 = yes	1 = 48.6	–
12	Stroke or cerebral vascular disease	0 = no/1 = yes	1 = 4.7	–
13	Diabetes or high blood sugar	0 = no/1 = yes	1 = 19.5	–
14	Chronic lung disease such as chronic bronchitis or emphysema	0 = no/1 = yes	1 = 9.9	–
15	Cancer or malignant tumor, including leukemia or lymphoma	0 = no/1 = yes	1 = 5.6	–
16	Arthritis, including osteoarthritis, or rheumatism	0 = no/1 = yes	1 = 27.0	–
17	Chronic renal disease	0 = no/1 = yes	1 = 2.8	–
18	Alzheimer's disease, dementia or any other serious memory impairment	0 = no/1 = yes	1 = 3.1	–
19	Difficulty getting dressed	0 = no/1 = yes	1 = 12.2	–
20	Difficulty walking across room	0 = no/1 = yes	1 = 8.0	0.5
21	Difficulty bathing/showering	0 = no/1 = yes	1 = 9.9	–
22	Difficulty eating	0 = no/1 = yes	1 = 3.8	–
23	Difficulty going in/out of bed	0 = no/1 = yes	1 = 7.5	–
24	Difficulty using toilet	0 = no/1 = yes	1 = 3.5	–
25	Difficulty preparing a warm meal	0 = no/1 = yes	1 = 5.4	0.5
26	Difficulty shopping groceries	0 = no/1 = yes	1 = 11.8	0.7
27	Difficulty using telephone	0 = no/1 = yes	1 = 1.6	–
28	Difficulty taking medicine	0 = no/1 = yes	1 = 1.9	1.6
29	Difficulty walking 100 meters	0 = no/1 = yes	1 = 12.5	0.7
30	Difficulty taking one flight of stairs	0 = no/1 = yes	1 = 23.6	0.7
31	Difficulty reaching or extending your arms above shoulder level	0 = no/1 = yes	1 = 14.8	–
32	Difficulty lifting or carrying weights over 10 pounds/5 kilos, like a heavy bag of groceries	0 = no/1 = yes	1 = 27.8	0.5
33	Difficulty concentrating	0 = never/rarely<br>0.5 = sometimes<br>1 = often/always	0 = 71.4<br>0.5 = 25.8<br>1 = 2.8	–
34	Everything takes effort	0 = never/rarely<br>0.5 = sometimes<br>1 = often/always	0 = 66.9<br>0.5 = 23.9<br>1 = 9.2	–
35	Sleep problems	0 = never/rarely<br>0.5 = sometimes<br>1 = often/always	0 = 52.7<br>0.5 = 35.3<br>1 = 12.0	0.2
36	Could not get going	0 = never/rarely<br>0.5 = sometimes<br>1 = often/always	0 = 65.7<br>0.5 =27.7<br>1 = 6.6	–
37	Poor appetite	0 = never/rarely<br>0.5 = sometimes<br>1 = often/always	0 = 89.0<br>0.5 = 8.0<br>1 = 3.1	–

Supplementary Table 2: University of California, Los Angeles – Loneliness Scale

**Please tell me how often the following applied to you in the last two weeks:**		rarely or never	sometimes	often or always	don't know/can't say
1	I felt like I was lacking companionship	(1)	(2)	(3)	(999)
2	I felt left out	(1)	(2)	(3)	(999)
3	I felt isolated from others	(1)	(2)	(3)	(999)

Supplementary Methods 2: Model selection

To study individual change over time, two frameworks exist: the mixed-effects (ME) approach and the latent-curve (LC) approach. Previous methodological research has shown that these two modeling frameworks share some overlap and estimates align with optimal data (see for instance McNeish and Matta, 2018 for a brief overview).

Since our analytical focus lies on the decomposition of within- and between-person effects in short-term dynamics (i.e., over weeks and months) between frailty and loneliness, a "complex" residual structure is necessary. In other words, we aim to examine the 1) separate autocorrelation of frailty and loneliness, 2) (within-person) cross-lagged effect of frailty on loneliness (i. e., frailty levels that are higher/lower than usual for a person at time t1 predict corresponding levels of loneliness that are higher/lower than usual at time t2) and loneliness on frailty, and 3) (within-person) within-time relationship between frailty and loneliness (i. e., frailty levels that are higher/lower than usual for a person at time t1 are associated with loneliness levels that are higher/lower than usual at time t1). While both ME and LC frameworks allow the specification of residual structures, the ME framework is often constrained by preprogrammed software options, whereas the LC framework allows the definition of any structure (McNeish & Matta, 2018). Therefore, we used a latent curve model with structured residuals (LCM-SR; Curran et al., 2014) to separate between-person from within-person variability.

Supplementary Figure 2: Pairwise correlations

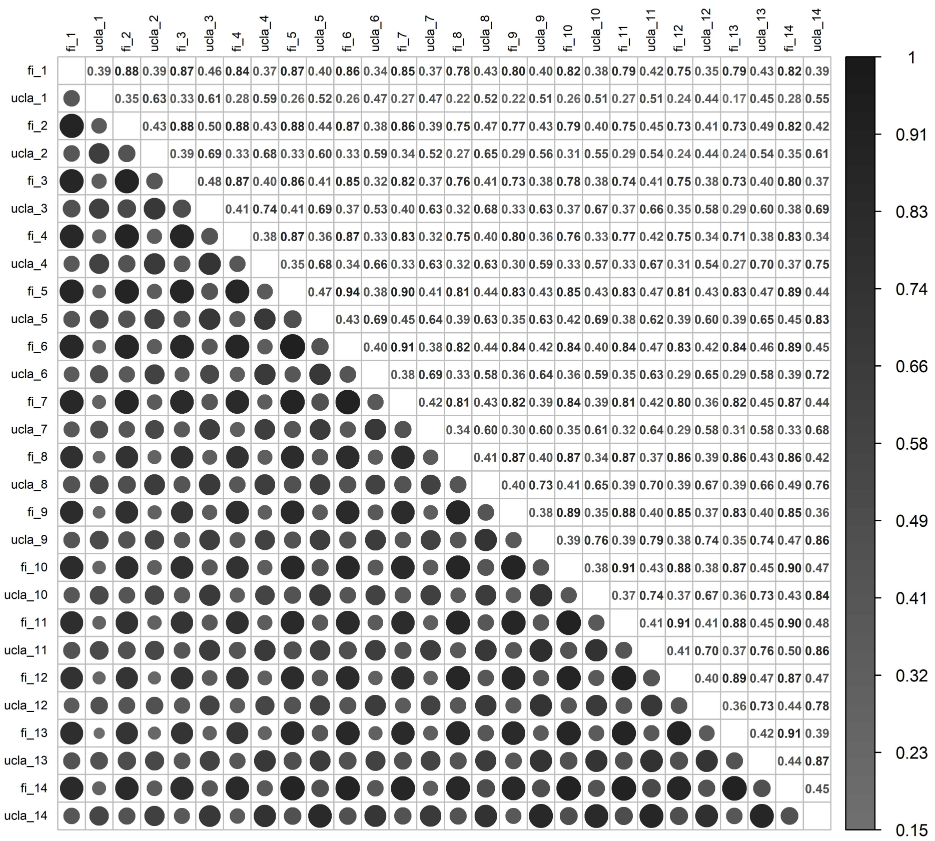

Supplementary Table 3: Parameters of FI models 1, 2, and 3

Parameter	model 1 Est.[95%CI]	model 2 Est.[95%CI]	model 3 Est.[95%CI]
Random effects: Means			
Intercept FI1*	0.18 [0.17, 0.19]	0.18 [0.17, 0.19]	0.18 [0.17, 0.19]
Intercept FI2*	0.20 [0.19, 0.21]	0.20 [0.19, 0.22]	0.20 [0.19, 0.22]
Slope FI1*	–	–	–0.00 [–0.00, 0.00]
Slope FI2*	–	–	0.00 [–0.00, 0.00]
Random Effects: Correlation (ζ)			
Intercept FI1 ↔ Intercept FI2	–	0.94 [0.92, 0.97]	0.95 [0.93, 0.97]
Autoregressive (FI → FI)			
α_1	0.89 [0.86, 0.92]	0.26 [0.12, 0.40]	0.26 [0.12, 0.40]
α_2	0.89 [0.84, 0.93]	0.31 [0.17, 0.45]	0.31 [0.17, 0.45]
α_3	0.87 [0.82, 0.93]	0.29 [0.05, 0.54]	0.29 [0.05, 0.54]
α_4	0.88 [0.83, 0.93]	0.08 [–0.10, 0.25]	0.08 [–0.10, 0.25]
α_5	0.94 [0.93, 0.96]	0.27 [0.09, 0.45]	0.27 [0.09, 0.45]
α_6	0.92 [0.89, 0.94]	0.20 [0.02, 0.37]	0.20 [0.02, 0.37]
α_7	0.84 [0.80, 0.88]	0.14 [–0.12, 0.39]	0.14 [–0.12, 0.39]
α_8	0.89 [0.85, 0.93]	0.22 [0.06, 0.38]	0.22 [0.06, 0.38]
α_9	0.91 [0.87, 0.94]	0.17 [–0.02, 0.37]	0.17 [–0.03, 0.37]
α_{10}	0.92 [0.90, 0.95]	0.10 [–0.23, 0.44]	0.10 [–0.24, 0.44]
α_{11}	0.93 [0.90, 0.96]	0.29 [0.07, 0.52]	0.29 [0.07, 0.51]
α_{12}	0.90 [0.85, 0.95]	0.22 [0.03, 0.41]	0.22 [0.03, 0.41]
α_{13}	0.92 [0.89, 0.96]	0.35 [0.10, 0.60]	0.35 [0.11, 0.60]

Note. FI = Frailty Index. In model 1 only fixed intercepts are specified. For models 2 and 3 random intercepts and fixed slopes are added, respectively. We report standardized parameter estimates and 95%-CI for all variables, except for variables with * in superscript. Here, unstandardized estimates are reported instead.

Supplementary Table 4: Model fit statistics and comparison of FI-models 1, 2, and 3

model	$\chi^2(df)$	$p - value$	ref.	$\Delta_{\chi^2}(\Delta df)$	$\Delta p - value$	TLI	CFI	SRMR	RMSEA [90% CI]	AIC	BIC
1	1227.65(90)	<.001	–	–	–	0.873	0.874	0.268	0.190 [0.177, 0.204]	–13192	–13074
2	230.76(87)	<.001	1	109.07(3)	<.001	0.983	0.983	0.034	0.070 [0.055, 0.086]	–14183	–14053
3	230.60(85)	<.001	2	0.076(2)	0.931	0.982	0.983	0.034	0.072 [0.056, 0.087]	–14179	–14041

Note. χ^2 = model fit statistic; df = degrees of freedom, ref. = reference model; Δ_{χ^2} = Satorra-Bentler scaled chi-square difference test; Δdf = differences in degrees of freedom; TLI = robust Tucker-Lewis-index; CFI = robust comparative-fit-index; SRMR = standardized root mean square residual; RMSEA = robust root mean square error of approximation; CI = confidence interval; AIC = Akaike information criterion; BIC = sample-size adjusted Bayesian information criterion.

Supplementary Table 5: Parameters of LS-models 1, 2, 3, and 4

	model 1	model 2	model 3	model 4
Parameter	Est.[95%CI]	Est.[95%CI]	Est.[95%CI]	Est.[95%CI]
Random effects: Means				
Intercept LS1*	3.46 [3.37, 3.54]	3.45 [3.36, 3.53]	3.39 [3.30, 3.48]	3.39 [3.30, 3.49]
Intercept LS2*	3.41 [3.33, 3.50]	3.48 [3.39, 3.58]	3.51 [3.41, 3.61]	3.51 [3.41, 3.61]
Slope LS1	–	–	0.02 [0.00, 0.03]	0.02 [0.00, 0.03]
Slope LS2	–	–	–0.01 [–0.02, 0.00]	–0.01 [–0.02, 0.00]
Random Effects: Correlation (ζ)				
Intercept LS1 $\leftrightarrow$ Intercept LS2	–	0.92 [0.86, 0.98]	0.92 [0.86, 0.98]	0.83 [0.71, 0.96]
Intercept LS1 $\leftrightarrow$ Slope LS1	–	–	–	–0.14 [–0.55, 0.27]
Intercept LS2 $\leftrightarrow$ Slope LS1	–	–	–	0.28 [–0.16, 0.72]
Autoregressive (LS $\rightarrow$ LS)				
β_1	0.64 [0.52, 0.77]	0.25 [0.09, 0.42]	0.26 [0.09, 0.42]	0.13 [–0.13, 0.38]
β_2	0.70 [0.58, 0.82]	0.20 [–0.03, 0.43]	0.19 [–0.04, 0.42]	0.09 [–0.19, 0.36]
β_3	0.75 [0.62, 0.88]	0.18 [–0.11, 0.46]	0.17 [–0.12, 0.45]	0.13 [–0.11, 0.38]
β_4	0.71 [0.59, 0.83]	0.01 [–0.22, 0.23]	0.01 [–0.22, 0.24]	0.02 [–0.22, 0.26]
β_5	0.69 [0.57, 0.81]	0.15 [–0.08, 0.39]	0.15 [–0.09, 0.38]	0.12 [–0.20, 0.43]
β_6	0.69 [0.60, 0.78]	0.27 [0.09, 0.44]	0.25 [0.08, 0.43]	0.18 [–0.07, 0.42]
β_7	0.61 [0.49, 0.72]	0.01 [–0.25, 0.27]	–0.01 [–0.27, 0.25]	0.01 [–0.27, 0.29]
β_8	0.74 [0.64, 0.84]	0.08 [–0.16, 0.31]	0.08 [–0.16, 0.31]	0.08 [–0.15, 0.31]
β_9	0.77 [0.66, 0.87]	0.06 [–0.24, 0.36]	0.06 [–0.23, 0.36]	0.07 [–0.22, 0.35]
β_{10}	0.75 [0.64, 0.85]	0.04 [–0.23, 0.30]	0.03 [–0.23, 0.30]	0.03 [–0.24, 0.30]
β_{11}	0.74 [0.64, 0.84]	–0.12 [–0.39, 0.15]	–0.12 [–0.39, 0.15]	–0.12 [–0.39, 0.15]
β_{12}	0.76 [0.66, 0.85]	0.05 [–0.19, 0.28]	0.04 [–0.20, 0.28]	0.04 [–0.20, 0.27]
β_{13}	0.86 [0.78, 0.94]	0.35 [0.09, 0.62]	0.35 [0.08, 0.62]	0.35 [0.08, 0.62]

Note. LS = loneliness. In model 1 only fixed intercepts are specified. For models 2 and 3 random intercepts and fixed slopes are added, respectively. In model 4, a random and a fixed slope are specified. We report standardized parameter estimates and 95%-CI for all variables, except for variables with * in superscript. Here, unstandardized estimates are reported instead.

Supplementary Table 6: Model fit statistics and comparison of LS models 1, 2, 3, and 4

model		$\chi^2(df)$	$p - value$	ref.	$\Delta_{\chi^2}(\Delta df)$	$\Delta p - value$	TLI	CFI	SRMR	RMSEA [90% CI]	AIC	BIC
1	1	1252.11(90)	<.001	–	–	–	0.763	0.766	0.383	0.187 [0.169, 0.205]	11231	11348
2	2	297.02(87)	<.001	1	443.67(3)	<.001	0.987	0.988	0.060	0.043 [0.000, 0.072]	10282	10411
3	3	287.94(85)	<.001	2	9.58 (2)	.008	0.988	0.989	0.059	0.041 [0.000, 0.071]	10276	10414
4	4	273.03(82)	<.001	3	4 (3)	.261	0.989	0.990	0.049	0.040 [0.000, 0.071]	10268	10418

Note. χ^2 = model fit statistic; *df* = degrees of freedom, ref. = reference model; Δ_{χ^2} = Satorra-Bentler scaled chi-square difference test; Δdf = differences in degrees of freedom; TLI = robust Tucker-Lewis-index; CFI = robust comparative-fit-index; SRMR = standardized root mean square residual; RMSEA = robust root mean square error of approximation; CI = confidence interval; AIC = Akaike information criterion; BIC = sample-size adjusted Bayesian information criterion.

Supplementary Table 7: Multiple group analysis: Sex (female vs. male)

	Female	Male
	n = 275	n = 151
Parameter	Est.[95%CI]	Est.[95%CI]
Random effects: Means		
Intercept FI1*	0.20 [0.18, 0.21]	0.15 [0.14, 0.17]
Intercept FI2*	0.22 [0.20, 0.24]	0.17 [0.15, 0.19]
Intercept LS1*	3.46 [3.34, 3.58]	3.27 [3.15, 3.38]
Intercept LS2*	3.59 [3.45, 3.73]	3.35 [3.24, 3.46]
Fixed effects: Means		
Slope LS1*	0.02 [0.00, 0.04]	0.01 [−0.01, 0.03]
Slope LS2*	−0.01 [−0.03, 0.00]	−0.00 [−0.02, 0.01]
Random Effects: Correlation		
ζ_1: Intercept FI1 ↔ Intercept FI2	0.94 [0.90, 0.97]	0.96 [0.93, 0.98]
ζ_2: Intercept FI1 ↔ Intercept LS1	0.54 [0.40, 0.67]	0.65 [0.49, 0.82]
ζ_3: Intercept FI1 ↔ Intercept LS2	0.56 [0.42, 0.69]	0.64 [0.48, 0.81]
ζ_4: Intercept FI2 ↔ Intercept LS1	0.49 [0.35, 0.64]	0.57 [0.41, 0.72]
ζ_5: Intercept FI2 ↔ Intercept LS2	0.50 [0.34, 0.65]	0.60 [0.46, 0.74]
ζ_6: Intercept LS1 ↔ Intercept LS2	0.91 [0.83, 0.98]	0.95 [0.86, 1.03]
Autoregressive (FI → FI)		
α_1	0.30 [0.11, 0.50]	0.16 [−0.08, 0.41]
α_2	0.30 [0.09, 0.50]	0.30 [0.14, 0.46]
α_3	0.35 [0.03, 0.66]	0.15 [−0.16, 0.47]
α_4	0.07 [−0.14, 0.29]	0.05 [−0.35, 0.45]
α_5	0.31 [0.10, 0.52]	0.19 [−0.22, 0.60]
α_6	0.27 [0.08, 0.46]	0.04 [−0.32, 0.40]
α_7	0.21 [−0.08, 0.49]	−0.19 [−0.47, 0.10]
α_8	0.26 [0.10, 0.43]	0.00 [−0.38, 0.38]
α_9	0.29 [0.09, 0.50]	−0.17 [−0.63, 0.29]
α_{10}	−0.13 [−0.41, 0.14]	0.46 [−0.06, 0.99]
α_{11}	0.25 [−0.02, 0.51]	0.38 [0.07, 0.68]
α_{12}	0.22 [−0.05, 0.50]	0.33 [0.02, 0.64]
α_{13}	0.33 [0.06, 0.59]	0.46 [0.04, 0.87]
Autoregressive (LS → LS)		
β_1	0.23 [0.05, 0.42]	0.29 [0.05, 0.53]
β_2	0.18 [−0.09, 0.45]	0.12 [−0.18, 0.42]
β_3	0.07 [−0.25, 0.40]	0.24 [−0.15, 0.63]
β_4	0.02 [−0.34, 0.38]	−0.13 [−0.54, 0.27]
β_5	0.11 [−0.22, 0.44]	0.15 [−0.19, 0.50]
β_6	0.24 [0.03, 0.44]	0.37 [0.02, 0.72]
β_7	0.08 [−0.22, 0.38]	−0.44 [−0.74, −0.14]
β_8	0.18 [−0.08, 0.43]	−0.24 [−0.60, 0.13]
β_9	−0.04 [−0.35, 0.28]	0.14 [−0.25, 0.54]
β_{10}	−0.03 [−0.31, 0.26]	0.04 [−0.43, 0.52]
β_{11}	−0.09 [−0.44, 0.25]	−0.16 [−0.42, 0.10]
β_{12}	−0.02 [−0.29, 0.25]	0.11 [−0.35, 0.57]
β_{13}	0.18 [−0.25, 0.61]	0.43 [0.15, 0.71]
Cross-lagged (LS → FI)		
δ_1	0.02 [−0.16, 0.21]	−0.03 [−0.32, 0.25]
δ_2	0.08 [−0.08, 0.24]	0.14 [−0.03, 0.31]
δ_3	0.07 [−0.11, 0.24]	−0.00 [−0.25, 0.25]
δ_4	0.11 [−0.15, 0.37]	−0.31 [−0.51, −0.12]
δ_5	−0.01 [−0.21, 0.18]	−0.15 [−0.40, 0.10]
δ_6	−0.01 [−0.15, 0.14]	−0.08 [−0.34, 0.18]
δ_7	0.08 [−0.07, 0.22]	0.10 [−0.19, 0.39]
δ_8	0.05 [−0.16, 0.26]	−0.17 [−0.43, 0.09]
δ_9	0.09 [−0.16, 0.34]	0.04 [−0.24, 0.32]
δ_{10}	−0.13 [−0.40, 0.13]	0.05 [−0.15, 0.26]
δ_{11}	0.13 [−0.11, 0.36]	0.06 [−0.20, 0.31]
δ_{12}	0.07 [−0.18, 0.31]	−0.10 [−0.32, 0.11]
δ_{13}	0.02 [−0.22, 0.26]	0.04 [−0.11, 0.18]
Cross-lagged (FI → LS)		
γ_1	0.09 [−0.04, 0.22]	0.18 [0.02, 0.33]
γ_2	0.18 [0.01, 0.35]	0.03 [−0.31, 0.38]
γ_3	0.15 [−0.09, 0.40]	0.27 [0.11, 0.44]
γ_4	0.03 [−0.26, 0.32]	−0.26 [−0.47, −0.06]
γ_5	−0.00 [−0.23, 0.23]	−0.05 [−0.36, 0.25]
γ_6	−0.09 [−0.23, 0.05]	−0.09 [−0.32, 0.14]
γ_7	0.08 [−0.07, 0.23]	−0.11 [−0.33, 0.11]
γ_8	0.15 [−0.05, 0.34]	0.18 [−0.25, 0.62]
γ_9	−0.06 [−0.30, 0.18]	0.23 [−0.09, 0.54]
γ_{10}	0.18 [−0.08, 0.44]	−0.02 [−0.36, 0.32]
γ_{11}	0.20 [−0.08, 0.47]	0.22 [0.01, 0.44]
γ_{12}	−0.12 [−0.33, 0.10]	0.19 [−0.23, 0.62]
γ_{13}	0.34 [0.03, 0.65]	0.03 [−0.09, 0.15]
Within-time (FI ↔ LS)		
λ_1	0.21 [0.08, 0.34]	0.31 [0.10, 0.52]
λ_2	0.21 [0.08, 0.34]	0.02 [−0.25, 0.29]
λ_3	0.19 [0.06, 0.33]	0.15 [−0.02, 0.32]
λ_4	0.22 [0.01, 0.42]	0.08 [−0.11, 0.26]
λ_5	0.20 [−0.09, 0.50]	0.00 [−0.31, 0.32]
λ_6	0.15 [−0.01, 0.32]	0.30 [0.02, 0.57]
λ_7	0.22 [0.08, 0.37]	0.03 [−0.15, 0.21]
λ_8	0.10 [−0.07, 0.26]	−0.01 [−0.38, 0.36]
λ_9	0.11 [−0.10, 0.31]	−0.01 [−0.40, 0.39]
λ_{10}	0.06 [−0.18, 0.31]	0.23 [−0.02, 0.49]
λ_{11}	0.12 [−0.15, 0.40]	0.14 [−0.03, 0.30]
λ_{12}	−0.13 [−0.30, 0.05]	0.46 [0.04, 0.87]
λ_{13}	0.12 [−0.06, 0.31]	−0.02 [−0.17, 0.13]
λ_{14}	0.11 [−0.09, 0.32]	0.10 [−0.12, 0.31]

Note. We report standardized parameter estimates and 95.00 % CI intervals for all variables, except for the means of fixed/random effects; here, we report unstandardized estimates, as indicated by *.

Model fit: $\chi^2(648)$ = 1461.66 p <.001; robust TLI = 0.928; robust CFI = 0.938; SRMR = 0.069; robust RMSEA [90% CI] = 0.088 [0.077, 0.098].

Supplementary Table 8: Multiple group analysis: Age ($\leq$ 74 vs. 75–79 vs. $\geq$ 80)

	$\leq$ 74<br>n = 166	75–79<br>n = 127	$\geq$ 80<br>n = 133
Parameter	Est.[95%CI]	Est.[95%CI]	Est.[95%CI]
Random effects: Means			
Intercept FI1*	0.14 [0.12, 0.15]	0.18 [0.16, 0.20]	0.24 [0.21, 0.27]
Intercept FI2*	0.15 [0.13, 0.17]	0.21 [0.18, 0.23]	0.27 [0.24, 0.29]
Intercept LS1*	3.30 [3.19, 3.41]	3.35 [3.17, 3.52]	3.57 [3.37, 3.76]
Intercept LS2*	3.31 [3.20, 3.42]	3.40 [3.26, 3.54]	3.82 [3.58, 4.05]
Fixed effects: Means			
Slope LS1*	0.01 [−0.01, 0.02]	0.02 [−0.01, 0.05]	0.03 [−0.00, 0.06]
Slope LS2*	0.00 [−0.01, 0.02]	0.00 [−0.02, 0.03]	−0.02 [−0.04, 0.00]
Random Effects: Correlation			
ζ_1: Intercept FI1 ↔ Intercept FI2	0.91 [0.86, 0.96]	0.95 [0.91, 0.98]	0.95 [0.91, 0.99]
ζ_2: Intercept FI1 ↔ Intercept LS1	0.59 [0.45, 0.73]	0.66 [0.47, 0.86]	0.49 [0.30, 0.68]
ζ_3: Intercept FI1 ↔ Intercept LS2	0.54 [0.38, 0.69]	0.54 [0.30, 0.79]	0.55 [0.37, 0.74]
ζ_4: Intercept FI2 ↔ Intercept LS1	0.48 [0.32, 0.65]	0.58 [0.41, 0.75]	0.45 [0.25, 0.66]
ζ_5: Intercept FI2 ↔ Intercept LS2	0.45 [0.27, 0.63]	0.50 [0.28, 0.71]	0.53 [0.32, 0.73]
ζ_6: Intercept LS1 ↔ Intercept LS2	0.93 [0.87, 0.99]	0.90 [0.73, 1.07]	0.94 [0.86, 1.01]
Autoregressive (FI → FI)			
α_1	0.31 [0.08, 0.54]	0.11 [−0.23, 0.44]	0.35 [0.08, 0.62]
α_2	0.32 [0.14, 0.50]	0.21 [−0.07, 0.50]	0.39 [0.06, 0.71]
α_3	0.40 [−0.04, 0.85]	0.37 [0.00, 0.73]	0.29 [−0.12, 0.70]
α_4	0.01 [−0.52, 0.54]	0.30 [0.02, 0.58]	0.11 [−0.23, 0.45]
α_5	0.28 [0.00, 0.55]	0.29 [0.01, 0.58]	0.33 [0.02, 0.65]
α_6	0.06 [−0.25, 0.36]	0.26 [−0.02, 0.54]	0.24 [−0.04, 0.52]
α_7	−0.04 [−0.31, 0.23]	0.35 [−0.18, 0.88]	0.00 [−0.34, 0.35]
α_8	0.24 [0.01, 0.47]	0.08 [−0.23, 0.40]	0.25 [−0.03, 0.54]
α_9	0.36 [0.16, 0.56]	0.15 [−0.24, 0.53]	−0.05 [−0.43, 0.34]
α_{10}	−0.27 [−0.62, 0.08]	0.47 [−0.00, 0.93]	−0.10 [−0.57, 0.36]
α_{11}	0.34 [−0.00, 0.67]	0.18 [−0.20, 0.56]	0.44 [0.12, 0.75]
α_{12}	0.24 [−0.25, 0.73]	0.17 [−0.10, 0.43]	0.18 [−0.10, 0.47]
α_{13}	0.25 [−0.06, 0.56]	0.35 [−0.03, 0.74]	0.50 [0.18, 0.83]
Autoregressive (LS → LS)			
β_1	0.07 [−0.36, 0.49]	0.13 [−0.05, 0.30]	0.49 [0.27, 0.70]
β_2	0.00 [−0.27, 0.27]	−0.18 [−0.56, 0.19]	0.53 [0.25, 0.80]
β_3	0.35 [0.07, 0.64]	−0.14 [−0.71, 0.43]	0.25 [−0.02, 0.52]
β_4	0.09 [−0.21, 0.38]	0.09 [−0.29, 0.47]	0.07 [−0.31, 0.44]
β_5	0.02 [−0.26, 0.30]	0.13 [−0.23, 0.50]	0.29 [−0.08, 0.65]
β_6	0.29 [−0.02, 0.60]	0.37 [0.02, 0.72]	0.23 [−0.07, 0.54]
β_7	0.21 [−0.32, 0.73]	−0.19 [−0.54, 0.17]	0.05 [−0.31, 0.41]
β_8	−0.02 [−0.56, 0.52]	0.06 [−0.38, 0.50]	−0.03 [−0.31, 0.25]
β_9	−0.44 [−0.94, 0.06]	−0.20 [−0.73, 0.34]	0.37 [−0.05, 0.79]
β_{10}	0.15 [−0.13, 0.43]	0.00 [−0.62, 0.62]	0.00 [−0.44, 0.44]
β_{11}	0.10 [−0.33, 0.53]	−0.23 [−0.68, 0.23]	−0.07 [−0.60, 0.46]
β_{12}	0.08 [−0.49, 0.64]	−0.08 [−0.38, 0.22]	0.27 [−0.17, 0.71]
β_{13}	0.36 [0.14, 0.58]	0.32 [−0.36, 1.00]	0.47 [−0.07, 1.00]
Cross-lagged (LS → FI)			
δ_1	−0.05 [−0.31, 0.21]	0.05 [−0.26, 0.36]	−0.02 [−0.23, 0.18]
δ_2	−0.03 [−0.21, 0.14]	0.19 [−0.08, 0.45]	0.21 [0.04, 0.38]
δ_3	−0.04 [−0.30, 0.22]	0.09 [−0.26, 0.43]	−0.01 [−0.24, 0.22]
δ_4	−0.05 [−0.35, 0.26]	−0.12 [−0.36, 0.12]	0.14 [−0.17, 0.44]
δ_5	0.11 [−0.07, 0.28]	−0.11 [−0.41, 0.19]	−0.17 [−0.43, 0.08]
δ_6	0.14 [−0.09, 0.36]	−0.03 [−0.33, 0.27]	−0.12 [−0.31, 0.08]
δ_7	0.07 [−0.08, 0.23]	0.01 [−0.16, 0.18]	0.11 [−0.16, 0.38]
δ_8	−0.06 [−0.19, 0.07]	0.17 [−0.13, 0.48]	−0.10 [−0.41, 0.20]
δ_9	−0.10 [−0.29, 0.09]	−0.19 [−0.45, 0.07]	0.25 [−0.16, 0.66]
δ_{10}	−0.09 [−0.37, 0.18]	−0.04 [−0.23, 0.15]	−0.07 [−0.81, 0.67]
δ_{11}	0.01 [−0.19, 0.22]	0.29 [−0.01, 0.60]	−0.12 [−0.37, 0.14]
δ_{12}	0.27 [−0.06, 0.61]	−0.33 [−0.60, −0.07]	0.14 [−0.14, 0.42]
δ_{13}	0.23 [0.06, 0.40]	0.18 [−0.12, 0.48]	−0.08 [−0.45, 0.28]
Cross-lagged (FI → LS)			
γ_1	0.15 [−0.02, 0.32]	0.13 [−0.13, 0.39]	−0.00 [−0.14, 0.13]
γ_2	0.20 [−0.07, 0.46]	0.38 [−0.02, 0.77]	0.04 [−0.17, 0.26]
γ_3	0.20 [−0.09, 0.49]	0.07 [−0.21, 0.36]	0.33 [0.01, 0.66]
γ_4	−0.06 [−0.25, 0.14]	0.16 [−0.21, 0.53]	−0.30 [−0.61, 0.01]
γ_5	0.09 [−0.22, 0.40]	−0.22 [−0.60, 0.16]	0.13 [−0.19, 0.45]
γ_6	−0.09 [−0.35, 0.17]	−0.01 [−0.25, 0.24]	−0.27 [−0.46, -0.08]
γ_7	−0.06 [−0.23, 0.11]	0.14 [−0.06, 0.33]	0.04 [−0.24, 0.32]
γ_8	0.04 [−0.14, 0.22]	0.01 [−0.26, 0.29]	0.17 [−0.18, 0.51]
γ_9	−0.05 [−0.36, 0.25]	−0.11 [−0.42, 0.21]	−0.00 [−0.42, 0.42]
γ_{10}	−0.09 [−0.32, 0.14]	0.17 [−0.10, 0.44]	0.01 [−0.57, 0.58]
γ_{11}	0.24 [−0.09, 0.56]	−0.04 [−0.31, 0.22]	0.36 [−0.10, 0.82]
γ_{12}	0.22 [−0.08, 0.51]	0.08 [−0.25, 0.42]	−0.12 [−0.43, 0.20]
γ_{13}	0.55 [0.24, 0.86]	0.08 [−0.48, 0.65]	0.15 [−0.12, 0.43]
Within-time (FI ↔ LS)			
λ_1	0.06 [−0.17, 0.29]	0.25 [0.07, 0.43]	0.38 [0.20, 0.57]
λ_2	0.08 [−0.16, 0.31]	0.34 [0.14, 0.53]	0.10 [−0.07, 0.26]
λ_3	0.20 [0.03, 0.37]	0.24 [−0.04, 0.52]	0.17 [−0.02, 0.35]
λ_4	−0.09 [−0.30, 0.13]	0.09 [−0.16, 0.34]	0.21 [−0.04, 0.46]
λ_5	0.20 [−0.04, 0.43]	0.14 [−0.25, 0.54]	0.13 [−0.14, 0.41]
λ_6	0.26 [0.01, 0.51]	0.03 [−0.37, 0.44]	0.24 [−0.06, 0.54]
λ_7	0.04 [−0.11, 0.20]	0.19 [−0.06, 0.44]	0.22 [0.02, 0.42]
λ_8	0.16 [−0.01, 0.33]	0.09 [−0.13, 0.32]	−0.01 [−0.27, 0.25]
λ_9	−0.15 [−0.33, 0.03]	−0.03 [−0.31, 0.26]	0.14 [−0.26, 0.53]
λ_{10}	−0.30 [−0.58, −0.03]	0.11 [−0.14, 0.36]	0.05 [−0.38, 0.47]
λ_{11}	0.21 [−0.19, 0.62]	−0.06 [−0.25, 0.13]	0.07 [−0.42, 0.55]
λ_{12}	0.28 [0.04, 0.51]	−0.17 [−0.41, 0.07]	0.13 [−0.50, 0.76]
λ_{13}	0.03 [−0.14, 0.20]	0.26 [−0.01, 0.53]	0.13 [−0.04, 0.31]
λ_{14}	0.29 [0.08, 0.51]	0.05 [−0.32, 0.43]	−0.04 [−0.42, 0.34]

Note. We report standardized parameter estimates and 95.00 % CI intervals for all variables, except for the means of fixed/random effects; here, we report unstandardized estimates, as indicated by *.
Model fit: $\chi^2(972)$ = 2100.69 p <.001; robust TLI = 0.944; robust CFI = 0.952; SRMR = 0.100; robust RMSEA [90% CI] = 0.076 [0.061, 0.089].

Supplementary Table 9: Multiple group analysis: Living alone (yes vs. no)

Parameter	Yes<br>n = 281<br>Est.[95%CI]	No<br>n = 145<br>Est.[95%CI]
Random effects: Means		
Intercept FI1*	0.19 [0.18, 0.21]	0.16 [0.14, 0.18]
Intercept FI2*	0.21 [0.20, 0.23]	0.18 [0.16, 0.21]
Intercept LS1*	3.48 [3.36, 3.60]	3.24 [3.11, 3.37]
Intercept LS2*	3.56 [3.43, 3.68]	3.41 [3.27, 3.56]
Fixed effects: Means		
Slope LS1*	0.01 [−0.01, 0.03]	0.02 [0.00, 0.04]
Slope LS2*	−0.01 [−0.02, 0.01]	−0.02 [−0.03, −0.00]
Random Effects: Correlation		
ζ_1: Intercept FI1 ↔ Intercept FI2	0.95 [0.92, 0.97]	0.94 [0.89, 0.99]
ζ_2: Intercept FI1 ↔ Intercept LS1	0.55 [0.42, 0.68]	0.59 [0.39, 0.78]
ζ_3: Intercept FI1 ↔ Intercept LS2	0.55 [0.41, 0.69]	0.65 [0.46, 0.84]
ζ_4: Intercept FI2 ↔ Intercept LS1	0.52 [0.38, 0.66]	0.48 [0.24, 0.72]
ζ_5: Intercept FI2 ↔ Intercept LS2	0.51 [0.36, 0.66]	0.52 [0.28, 0.76]
ζ_6: Intercept LS1 ↔ Intercept LS2	0.93 [0.86, 1.00]	0.95 [0.84, 1.06]
Autoregressive (FI → FI)		
α_1	0.26 [0.07, 0.45]	0.22 [−0.04, 0.48]
α_2	0.26 [0.09, 0.43]	0.37 [0.12, 0.63]
α_3	0.29 [0.02, 0.55]	0.28 [−0.21, 0.76]
α_4	0.16 [−0.02, 0.33]	−0.22 [−0.79, 0.34]
α_5	0.34 [0.15, 0.53]	0.09 [−0.22, 0.40]
α_6	0.24 [0.04, 0.44]	0.18 [−0.15, 0.52]
α_7	−0.05 [−0.23, 0.13]	0.36 [−0.03, 0.75]
α_8	0.17 [−0.03, 0.37]	0.27 [0.03, 0.50]
α_9	0.09 [−0.19, 0.38]	0.31 [−0.04, 0.66]
α_{10}	0.11 [−0.31, 0.53]	0.14 [−1.15, 1.43]
α_{11}	0.23 [−0.02, 0.49]	0.60 [0.30, 0.89]
α_{12}	0.22 [−0.03, 0.46]	0.44 [0.02, 0.85]
α_{13}	0.15 [−0.12, 0.43]	0.73 [0.51, 0.94]
Autoregressive (LS → LS)		
β_1	0.25 [0.08, 0.41]	0.22 [−0.16, 0.60]
β_2	0.24 [0.01, 0.48]	−0.08 [−0.57, 0.41]
β_3	0.08 [−0.23, 0.40]	0.36 [−0.07, 0.79]
β_4	0.07 [−0.23, 0.37]	−0.12 [−0.73, 0.49]
β_5	0.09 [−0.19, 0.36]	0.37 [−0.05, 0.78]
β_6	0.32 [0.13, 0.52]	0.09 [−0.33, 0.52]
β_7	0.01 [−0.30, 0.32]	−0.04 [−0.65, 0.58]
β_8	0.11 [−0.16, 0.38]	0.07 [−0.46, 0.61]
β_9	0.20 [−0.13, 0.53]	−0.28 [−0.87, 0.30]
β_{10}	0.01 [−0.24, 0.26]	0.08 [−0.57, 0.72]
β_{11}	−0.18 [−0.56, 0.20]	−0.10 [−0.48, 0.27]
β_{12}	0.02 [−0.32, 0.36]	−0.09 [−0.87, 0.69]
β_{13}	0.31 [0.00, 0.63]	0.33 [−0.54, 1.20]
Cross-lagged (LS → FI)		
δ_1	−0.06 [−0.21, 0.09]	0.22 [−0.24, 0.68]
δ_2	0.04 [−0.11, 0.18]	0.27 [0.01, 0.53]
δ_3	0.12 [−0.05, 0.29]	−0.24 [−0.53, 0.06]
δ_4	0.01 [−0.18, 0.19]	−0.11 [−0.53, 0.31]
δ_5	−0.06 [−0.24, 0.11]	−0.05 [−0.44, 0.35]
δ_6	−0.02 [−0.18, 0.14]	−0.08 [−0.27, 0.12]
δ_7	0.08 [−0.05, 0.21]	0.20 [−0.14, 0.54]
δ_8	−0.12 [−0.29, 0.05]	0.31 [−0.05, 0.67]
δ_9	−0.04 [−0.25, 0.17]	0.37 [−0.15, 0.88]
δ_{10}	0.01 [−0.22, 0.23]	−0.12 [−0.49, 0.26]
δ_{11}	0.08 [−0.10, 0.26]	0.15 [−0.15, 0.46]
δ_{12}	0.05 [−0.20, 0.31]	0.06 [−0.15, 0.28]
δ_{13}	0.15 [−0.03, 0.34]	−0.23 [−0.50, 0.04]
Cross-lagged (FI → LS)		
γ_1	0.12 [−0.01, 0.25]	0.06 [−0.15, 0.28]
γ_2	0.13 [−0.05, 0.31]	0.32 [−0.06, 0.69]
γ_3	0.16 [−0.04, 0.36]	0.28 [−0.07, 0.62]
γ_4	−0.09 [−0.32, 0.13]	0.11 [−0.52, 0.74]
γ_5	−0.03 [−0.25, 0.20]	−0.04 [−0.25, 0.17]
γ_6	−0.08 [−0.22, 0.06]	−0.11 [−0.33, 0.12]
γ_7	0.05 [−0.10, 0.20]	0.01 [−0.21, 0.24]
γ_8	0.05 [−0.16, 0.26]	0.17 [−0.27, 0.62]
γ_9	0.01 [−0.22, 0.24]	−0.05 [−0.47, 0.37]
γ_{10}	0.06 [−0.19, 0.30]	0.57 [−0.06, 1.20]
γ_{11}	0.28 [0.01, 0.55]	0.23 [−0.43, 0.89]
γ_{12}	0.13 [−0.12, 0.39]	−0.29 [−1.11, 0.52]
γ_{13}	0.05 [−0.15, 0.25]	0.35 [−0.21, 0.92]
Within-time (FI ↔ LS)		
λ_1	0.22 [0.09, 0.35]	0.29 [0.10, 0.48]
λ_2	0.17 [0.03, 0.32]	0.16 [−0.03, 0.36]
λ_3	0.20 [0.08, 0.32]	0.28 [0.02, 0.54]
λ_4	0.18 [−0.01, 0.37]	0.10 [−0.19, 0.38]
λ_5	0.23 [−0.04, 0.50]	0.05 [−0.42, 0.51]
λ_6	0.23 [0.04, 0.42]	−0.01 [−0.25, 0.22]
λ_7	0.25 [0.10, 0.40]	0.05 [−0.18, 0.28]
λ_8	0.03 [−0.15, 0.21]	0.28 [−0.02, 0.58]
λ_9	0.06 [−0.14, 0.27]	−0.01 [−0.48, 0.46]
λ_{10}	0.08 [−0.10, 0.27]	0.15 [−0.44, 0.74]
λ_{11}	0.14 [−0.07, 0.36]	0.38 [−0.22, 0.98]
λ_{12}	0.23 [−0.10, 0.57]	−0.21 [−0.45, 0.04]
λ_{13}	0.18 [0.02, 0.34]	−0.01 [−0.19, 0.18]
λ_{14}	0.08 [−0.09, 0.25]	0.26 [−0.38, 0.89]

Note. We report standardized parameter estimates and 95.00 % CI intervals for all variables, except for the means of fixed/random effects; here, we report unstandardized estimates, as indicated by *.

Model fit: $\chi^2(648)$ = 1633.09 p <.001; robust TLI = 0.953; robust CFI = 0.960; SRMR = 0.082; robust RMSEA [90% CI] = 0.071 [0.057, 0.085].

Supplementary Table 10: Multiple group analysis: social participation (yes vs. no; participation in at least one social activity)

	Yes n = 249	No n = 177
Parameter	Est.[95%CI]	Est.[95%CI]
Random effects: Means		
Intercept FI1*	0.14 [0.13, 0.16]	0.21 [0.19, 0.23]
Intercept FI2*	0.17 [0.15, 0.18]	0.23 [0.21, 0.25]
Intercept LS1*	3.28 [3.19, 3.38]	3.47 [3.34, 3.61]
Intercept LS2*	3.33 [3.22, 3.44]	3.63 [3.49, 3.78]
Fixed effects: Means		
Slope LS1*	0.01 [−0.01, 0.02]	0.02 [0.00, 0.04]
Slope LS2*	−0.00 [−0.02, 0.01]	−0.01 [−0.03, 0.01]
Random Effects: Correlation		
ζ_1: Intercept FI1 ↔ Intercept FI2	0.94 [0.90, 0.97]	0.94 [0.90, 0.97]
ζ_2: Intercept FI1 ↔ Intercept LS1	0.44 [0.27, 0.61]	0.60 [0.48, 0.72]
ζ_3: Intercept FI1 ↔ Intercept LS2	0.42 [0.25, 0.59]	0.61 [0.48, 0.73]
ζ_4: Intercept FI2 ↔ Intercept LS1	0.37 [0.16, 0.58]	0.55 [0.42, 0.67]
ζ_5: Intercept FI2 ↔ Intercept LS2	0.37 [0.14, 0.59]	0.57 [0.44, 0.70]
ζ_6: Intercept LS1 ↔ Intercept LS2	0.95 [0.87, 1.03]	0.91 [0.84, 0.98]
Autoregressive (FI → FI)		
α_1	0.31 [0.08, 0.53]	0.23 [0.02, 0.43]
α_2	0.15 [−0.02, 0.32]	0.36 [0.17, 0.55]
α_3	0.17 [−0.04, 0.37]	0.34 [0.01, 0.68]
α_4	−0.06 [−0.37, 0.26]	0.15 [−0.07, 0.37]
α_5	0.33 [0.09, 0.56]	0.30 [0.06, 0.55]
α_6	0.25 [−0.01, 0.50]	0.24 [0.02, 0.46]
α_7	0.31 [−0.18, 0.80]	0.01 [−0.25, 0.26]
α_8	0.22 [−0.06, 0.50]	0.20 [0.02, 0.39]
α_9	0.27 [−0.04, 0.57]	0.08 [−0.14, 0.31]
α_{10}	0.29 [−0.22, 0.80]	−0.11 [−0.50, 0.28]
α_{11}	0.31 [−0.05, 0.67]	0.29 [0.01, 0.57]
α_{12}	0.24 [−0.07, 0.55]	0.23 [−0.01, 0.47]
α_{13}	0.36 [0.03, 0.69]	0.35 [0.04, 0.67]
Autoregressive (LS → LS)		
β_1	0.15 [−0.14, 0.44]	0.26 [0.09, 0.44]
β_2	0.30 [0.05, 0.55]	0.10 [−0.19, 0.38]
β_3	0.29 [0.01, 0.58]	0.05 [−0.32, 0.42]
β_4	0.04 [−0.19, 0.27]	0.02 [−0.30, 0.35]
β_5	0.12 [−0.07, 0.30]	0.14 [−0.17, 0.45]
β_6	0.38 [0.16, 0.60]	0.22 [−0.03, 0.46]
β_7	0.36 [0.04, 0.68]	−0.24 [−0.52, 0.04]
β_8	−0.07 [−0.47, 0.34]	0.08 [−0.21, 0.36]
β_9	0.12 [−0.37, 0.62]	0.02 [−0.38, 0.42]
β_{10}	0.27 [−0.20, 0.73]	−0.11 [−0.38, 0.16]
β_{11}	−0.01 [−0.43, 0.41]	−0.16 [−0.51, 0.20]
β_{12}	0.27 [−0.18, 0.72]	−0.11 [−0.45, 0.24]
β_{13}	0.23 [−0.08, 0.53]	0.52 [0.24, 0.80]
Cross-lagged (LS → FI)		
δ_1	−0.13 [−0.32, 0.07]	0.06 [−0.16, 0.28]
δ_2	0.15 [−0.02, 0.32]	0.06 [−0.11, 0.23]
δ_3	0.02 [−0.20, 0.25]	0.05 [−0.16, 0.25]
δ_4	−0.06 [−0.25, 0.13]	−0.04 [−0.24, 0.16]
δ_5	−0.01 [−0.21, 0.19]	−0.08 [−0.26, 0.10]
δ_6	−0.20 [−0.36, −0.03]	0.03 [−0.13, 0.19]
δ_7	0.13 [−0.06, 0.32]	0.02 [−0.16, 0.21]
δ_8	−0.07 [−0.25, 0.11]	0.05 [−0.17, 0.27]
δ_9	−0.03 [−0.27, 0.21]	0.07 [−0.27, 0.40]
δ_{10}	0.02 [−0.18, 0.22]	−0.05 [−0.32, 0.23]
δ_{11}	0.12 [−0.14, 0.38]	0.09 [−0.13, 0.31]
δ_{12}	0.19 [−0.08, 0.45]	−0.04 [−0.22, 0.14]
δ_{13}	0.16 [−0.07, 0.39]	0.02 [−0.21, 0.24]
Cross-lagged (FI → LS)		
γ_1	0.10 [−0.05, 0.25]	0.12 [−0.01, 0.25]
γ_2	0.03 [−0.14, 0.19]	0.21 [−0.04, 0.46]
γ_3	0.23 [0.06, 0.41]	0.12 [−0.14, 0.38]
γ_4	−0.15 [−0.34, 0.03]	−0.06 [−0.31, 0.19]
γ_5	−0.11 [−0.30, 0.08]	−0.03 [−0.29, 0.22]
γ_6	−0.05 [−0.21, 0.11]	−0.10 [−0.27, 0.07]
γ_7	0.01 [−0.21, 0.22]	0.08 [−0.08, 0.24]
γ_8	0.13 [−0.28, 0.54]	0.12 [−0.10, 0.33]
γ_9	0.10 [−0.14, 0.34]	−0.08 [−0.33, 0.16]
γ_{10}	0.02 [−0.19, 0.22]	0.18 [−0.25, 0.60]
γ_{11}	0.19 [−0.19, 0.57]	0.22 [−0.02, 0.46]
γ_{12}	−0.11 [−0.32, 0.10]	0.25 [−0.07, 0.58]
γ_{13}	0.30 [−0.04, 0.64]	0.03 [−0.12, 0.18]
Within-time (FI ↔ LS)		
λ_1	0.19 [−0.01, 0.38]	0.26 [0.12, 0.40]
λ_2	0.24 [0.05, 0.43]	0.14 [−0.01, 0.28]
λ_3	0.11 [−0.01, 0.23]	0.20 [0.04, 0.36]
λ_4	0.19 [0.01, 0.37]	0.13 [−0.11, 0.37]
λ_5	0.08 [−0.21, 0.37]	0.17 [−0.10, 0.43]
λ_6	0.19 [0.02, 0.37]	0.16 [−0.08, 0.41]
λ_7	0.27 [0.08, 0.47]	0.18 [0.03, 0.33]
λ_8	−0.02 [−0.31, 0.26]	0.11 [−0.07, 0.29]
λ_9	0.06 [−0.18, 0.30]	0.03 [−0.22, 0.29]
λ_{10}	−0.11 [−0.28, 0.06]	0.15 [−0.12, 0.42]
λ_{11}	0.09 [−0.16, 0.34]	0.15 [−0.16, 0.47]
λ_{12}	−0.02 [−0.28, 0.24]	0.17 [−0.21, 0.55]
λ_{13}	0.04 [−0.13, 0.21]	0.13 [−0.04, 0.31]
λ_{14}	0.12 [−0.16, 0.40]	0.08 [−0.09, 0.25]

Note. We report standardized parameter estimates and 95.00 % CI intervals for all variables, except for the means of fixed/random effects; here, we report unstandardized estimates, as indicated by *.

Model fit: $\chi^2(648)$ = 1343.61 p <.001; robust TLI = 0.953; robust CFI = 0.960; SRMR = 0.065; robust RMSEA [90% CI] = 0.069 [0.058, 0.058].

Supplementary Table 11: Analysis with `bedrest` as time-varying covariate ($N = 162$)

Parameter	Est.[95%CI]
Fixed/Random effects: Means	
Intercept FI1*	0.16 [0.14, 0.17]
Intercept FI2*	0.18 [0.16, 0.19]
Intercept LS1*	3.32 [3.19, 3.45]
Intercept LS2*	3.53 [3.37, 3.68]
Random effects: Means	
Slope LS1*	0.02 [−0.01, 0.05]
Slope LS2*	−0.02 [−0.03, −0.01]
Random Effects: Correlation	
Intercept FI1 ↔ Intercept FI2	0.96 [0.93, 0.98]
Intercept FI1 ↔ Intercept LS1	0.50 [0.30, 0.70]
Intercept FI1 ↔ Intercept LS2	0.52 [0.35, 0.69]
Intercept FI2 ↔ Intercept LS1	0.51 [0.31, 0.70]
Intercept FI2 ↔ Intercept LS2	0.53 [0.38, 0.69]
Intercept LS1 ↔ Intercept LS2	0.97 [0.88, 1.07]
Autoregressive (FI → FI)	
α_1	0.18 [0.01, 0.35]
α_2	0.21 [−0.01, 0.43]
α_3	0.27 [−0.02, 0.57]
α_4	0.14 [−0.16, 0.44]
α_5	0.27 [0.05, 0.49]
α_6	0.21 [−0.06, 0.48]
α_7	−0.02 [−0.23, 0.20]
α_8	0.15 [−0.09, 0.39]
α_9	0.34 [0.17, 0.52]
α_{10}	0.09 [−0.14, 0.33]
α_{11}	0.09 [−0.09, 0.27]
α_{12}	0.17 [−0.04, 0.39]
α_{13}	0.40 [0.16, 0.65]
Autoregressive (LS → LS)	
β_1	0.28 [0.01, 0.55]
β_2	0.51 [0.27, 0.76]
β_3	0.46 [0.14, 0.78]
β_4	0.47 [0.16, 0.79]
β_5	0.41 [0.16, 0.66]
β_6	0.36 [0.13, 0.59]
β_7	−0.07 [−0.42, 0.28]
β_8	−0.15 [−0.44, 0.14]
β_9	0.06 [−0.36, 0.48]
β_{10}	−0.20 [−0.51, 0.12]
β_{11}	−0.23 [−0.49, 0.03]
β_{12}	−0.04 [−0.34, 0.25]
β_{13}	0.22 [−0.08, 0.52]
Cross-lagged (LS → FI)	
δ_1	−0.05 [−0.22, 0.12]
δ_2	0.12 [−0.03, 0.27]
δ_3	0.07 [−0.13, 0.26]
δ_4	0.04 [−0.22, 0.30]
δ_5	0.03 [−0.16, 0.22]
δ_6	−0.00 [−0.25, 0.24]
δ_7	0.08 [−0.10, 0.27]
δ_8	0.09 [−0.25, 0.43]
δ_9	−0.01 [−0.22, 0.20]
δ_{10}	0.06 [−0.11, 0.22]
δ_{11}	0.06 [−0.10, 0.22]
δ_{12}	−0.04 [−0.20, 0.11]
δ_{13}	0.09 [−0.05, 0.24]
Cross-lagged (FI → LS)	
γ_1	0.04 [−0.11, 0.19]
γ_2	0.20 [−0.03, 0.43]
γ_3	0.24 [0.02, 0.45]
γ_4	−0.21 [−0.40, −0.01]
γ_5	0.15 [−0.05, 0.36]
γ_6	0.02 [−0.12, 0.16]
γ_7	0.15 [−0.11, 0.42]
γ_8	−0.27 [−0.49, −0.05]
γ_9	0.08 [−0.19, 0.35]
γ_{10}	0.11 [−0.17, 0.39]
γ_{11}	0.13 [−0.07, 0.32]
γ_{12}	−0.07 [−0.32, 0.19]
γ_{13}	0.12 [−0.07, 0.30]
Within-time (FI ↔ LS)	
λ_1	0.16 [−0.02, 0.34]
λ_2	0.10 [−0.09, 0.29]
λ_3	0.10 [−0.02, 0.23]
λ_4	0.25 [−0.08, 0.57]
λ_5	−0.11 [−0.34, 0.11]
λ_6	0.27 [0.09, 0.44]
λ_7	0.16 [−0.05, 0.38]
λ_8	−0.08 [−0.30, 0.14]
λ_9	0.17 [−0.09, 0.43]
λ_{10}	0.10 [−0.10, 0.31]
λ_{11}	−0.11 [−0.35, 0.12]
λ_{12}	0.15 [−0.19, 0.49]
λ_{13}	0.03 [−0.14, 0.20]
λ_{14}	0.09 [−0.06, 0.23]
Time-varying covariate	
$\text{bedrest}_1 \rightarrow \text{r\_fi}_1$	0.21 [0.06, 0.35]
$\text{bedrest}_1 \rightarrow \text{r\_ucla}_1$	−0.08 [−0.21, 0.04]
$\text{bedrest}_2 \rightarrow \text{r\_fi}_2$	0.42 [0.23, 0.60]
$\text{bedrest}_2 \rightarrow \text{r\_ucla}_2$	0.00 [−0.20, 0.20]
$\text{bedrest}_3 \rightarrow \text{r\_fi}_3$	0.32 [0.03, 0.60]
$\text{bedrest}_3 \rightarrow \text{r\_ucla}_3$	−0.13 [−0.29, 0.02]
$\text{bedrest}_4 \rightarrow \text{r\_fi}_4$	0.28 [0.10, 0.46]
$\text{bedrest}_4 \rightarrow \text{r\_ucla}_4$	−0.09 [−0.27, 0.09]
$\text{bedrest}_5 \rightarrow \text{r\_fi}_5$	0.39 [0.20, 0.58]
$\text{bedrest}_5 \rightarrow \text{r\_ucla}_5$	0.18 [−0.00, 0.37]
$\text{bedrest}_6 \rightarrow \text{r\_fi}_6$	0.29 [0.13, 0.46]
$\text{bedrest}_6 \rightarrow \text{r\_ucla}_6$	0.04 [−0.08, 0.17]
$\text{bedrest}_7 \rightarrow \text{r\_fi}_7$	0.24 [0.03, 0.46]
$\text{bedrest}_7 \rightarrow \text{r\_ucla}_7$	−0.02 [−0.14, 0.11]
$\text{bedrest}_8 \rightarrow \text{r\_fi}_8$	0.37 [0.18, 0.56]
$\text{bedrest}_8 \rightarrow \text{r\_ucla}_8$	−0.12 [−0.28, 0.05]
$\text{bedrest}_9 \rightarrow \text{r\_fi}_9$	0.30 [0.11, 0.50]
$\text{bedrest}_9 \rightarrow \text{r\_ucla}_9$	0.23 [0.05, 0.42]
$\text{bedrest}_{10} \rightarrow \text{r\_fi}_{10}$	0.33 [0.20, 0.47]
$\text{bedrest}_{10} \rightarrow \text{r\_ucla}_{10}$	−0.13 [−0.26, 0.00]
$\text{bedrest}_{11} \rightarrow \text{r\_fi}_{11}$	0.20 [0.02, 0.39]
$\text{bedrest}_{11} \rightarrow \text{r\_ucla}_{11}$	0.09 [−0.08, 0.27]
$\text{bedrest}_{12} \rightarrow \text{r\_fi}_{12}$	0.34 [0.15, 0.52]
$\text{bedrest}_{12} \rightarrow \text{r\_ucla}_{12}$	0.01 [−0.22, 0.23]
$\text{bedrest}_{13} \rightarrow \text{r\_fi}_{13}$	0.26 [0.11, 0.42]
$\text{bedrest}_{13} \rightarrow \text{r\_ucla}_{13}$	0.19 [−0.01, 0.39]
$\text{bedrest}_{14} \rightarrow \text{r\_fi}_{14}$	0.28 [0.12, 0.45]
$\text{bedrest}_{14} \rightarrow \text{r\_ucla}_{14}$	0.06 [−0.15, 0.27]

Note. We report standardized parameter estimates and 95.00 % CI intervals for all variables, except for the means of fixed/random effects; here, we report unstandardized estimates, as indicated by *. Model fit: $\chi^2(688) = 1195.14$ $p < .001$; robust TLI = 0.942; robust CFI = 0.948; SRMR = 0.148; robust RMSEA [90% CI] = 0.055 [0.047, 0.064].

Supplementary Table 12: Analysis with `falls` as time-varying covariate ($N = 162$)

Parameters	Est. [95%CI]
Random effects: Means	
Intercept FI1*	0.16 [0.15, 0.18]
Intercept FI2*	0.18 [0.16, 0.20]
Intercept LS1*	3.32 [3.19, 3.44]
Intercept LS2*	3.53 [3.38, 3.68]
Fixed effects: Means	
Slope LS1*	0.02 [–0.00, 0.05]
Slope LS2*	–0.02 [–0.03, –0.00]
Random Effects: Correlation	
Intercept FI1 ↔ Intercept FI2	0.96 [0.93, 0.99]
Intercept FI1 ↔ Intercept LS1	0.52 [0.30, 0.74]
Intercept FI1 ↔ Intercept LS2	0.54 [0.37, 0.71]
Intercept FI2 ↔ Intercept LS1	0.52 [0.33, 0.71]
Intercept FI2 ↔ Intercept LS2	0.55 [0.40, 0.71]
Intercept LS1 ↔ Intercept LS2	0.95 [0.84, 1.06]
Autoregressive (FI → FI)	
α_1	0.24 [0.04, 0.43]
α_2	0.30 [0.04, 0.56]
α_3	0.37 [0.04, 0.70]
α_4	0.18 [–0.20, 0.55]
α_5	0.22 [–0.07, 0.51]
α_6	0.19 [–0.08, 0.46]
α_7	-0.09 [–0.34, 0.16]
α_8	0.12 [–0.14, 0.38]
α_9	0.38 [0.17, 0.59]
α_{10}	0.09 [–0.18, 0.36]
α_{11}	0.18 [–0.02, 0.38]
α_{12}	0.24 [0.02, 0.46]
α_{13}	0.48 [0.23, 0.73]
Autoregressive (LS → LS)	
β_1	0.26 [–0.01, 0.54]
β_2	0.49 [0.23, 0.76]
β_3	0.44 [0.07, 0.80]
β_4	0.45 [0.12, 0.79]
β_5	0.40 [0.13, 0.66]
β_6	0.35 [0.11, 0.58]
β_7	–0.09 [–0.44, 0.26]
β_8	–0.19 [–0.49, 0.11]
β_9	0.05 [–0.39, 0.49]
β_{10}	–0.21 [–0.53, 0.11]
β_{11}	–0.24 [–0.53, 0.05]
β_{12}	–0.01 [–0.30, 0.29]
β_{13}	0.24 [–0.07, 0.55]
Cross-lagged (LS → FI)	
δ_1	–0.09 [–0.27, 0.10]
δ_2	0.12 [–0.01, 0.24]
δ_3	0.05 [–0.15, 0.25]
δ_4	0.07 [–0.21, 0.34]
δ_5	0.02 [–0.21, 0.26]
δ_6	–0.06 [–0.34, 0.22]
δ_7	0.07 [–0.12, 0.26]
δ_8	0.07 [–0.27, 0.41]
δ_9	–0.01 [–0.23, 0.22]
δ_{10}	0.03 [–0.17, 0.23]
δ_{11}	0.14 [–0.06, 0.34]
δ_{12}	–0.01 [–0.17, 0.15]
δ_{13}	0.13 [–0.00, 0.25]
Cross-lagged (FI → LS)	
γ_1	0.05 [–0.12, 0.22]
γ_2	0.19 [–0.02, 0.40]
γ_3	0.21 [–0.05, 0.46]
γ_4	–0.23 [–0.46, –0.00]
γ_5	0.07 [–0.18, 0.32]
γ_6	0.01 [–0.12, 0.14]
γ_7	0.20 [–0.07, 0.46]
γ_8	–0.24 [–0.50, 0.02]
γ_9	0.04 [–0.23, 0.30]
γ_{10}	0.09 [–0.19, 0.37]
γ_{11}	0.10 [–0.09, 0.29]
γ_{12}	–0.04 [–0.30, 0.23]
γ_{13}	0.14 [–0.03, 0.31]
Within-time (FI ↔ LS)	
λ_1	0.16 [–0.03, 0.35]
λ_2	0.09 [–0.11, 0.29]
λ_3	0.06 [–0.09, 0.20]
λ_4	0.20 [–0.15, 0.54]
λ_5	–0.06 [–0.28, 0.17]
λ_6	0.24 [0.06, 0.42]
λ_7	0.14 [–0.09, 0.37]
λ_8	–0.09 [–0.32, 0.14]
λ_9	0.22 [–0.03, 0.47]
λ_{10}	0.04 [–0.17, 0.25]
λ_{11}	–0.14 [–0.39, 0.10]
λ_{12}	0.15 [–0.21, 0.52]
λ_{13}	0.11 [–0.05, 0.26]
λ_{14}	0.10 [–0.02, 0.22]
Time-varying covariate	
$\text{falls}_{1} \rightarrow \text{r\_fi}_{1}$	0.34 [0.10, 0.59]
$\text{falls}_{1} \rightarrow \text{r\_ucla}_{1}$	0.01 [–0.07, 0.09]
$\text{falls}_{2} \rightarrow \text{r\_fi}_{2}$	0.16 [–0.09, 0.41]
$\text{falls}_{2} \rightarrow \text{r\_ucla}_{2}$	–0.02 [–0.06, 0.01]
$\text{falls}_{3} \rightarrow \text{r\_fi}_{3}$	–0.04 [–0.08, –0.00]
$\text{falls}_{3} \rightarrow \text{r\_ucla}_{3}$	–0.11 [–0.22, 0.00]
$\text{falls}_{4} \rightarrow \text{r\_fi}_{4}$	0.09 [–0.12, 0.31]
$\text{falls}_{4} \rightarrow \text{r\_ucla}_{4}$	–0.11 [–0.30, 0.08]
$\text{falls}_{5} \rightarrow \text{r\_fi}_{5}$	–0.04 [–0.11, 0.03]
$\text{falls}_{5} \rightarrow \text{r\_ucla}_{5}$	–0.02 [–0.04, –0.00]
$\text{falls}_{6} \rightarrow \text{r\_fi}_{6}$	0.08 [–0.01, 0.18]
$\text{falls}_{6} \rightarrow \text{r\_ucla}_{6}$	0.00 [–0.14, 0.14]
$\text{falls}_{7} \rightarrow \text{r\_fi}_{7}$	0.02 [–0.15, 0.19]
$\text{falls}_{7} \rightarrow \text{r\_ucla}_{7}$	–0.05 [–0.09, –0.00]
$\text{falls}_{8} \rightarrow \text{r\_fi}_{8}$	0.13 [–0.06, 0.33]
$\text{falls}_{8} \rightarrow \text{r\_ucla}_{8}$	–0.02 [–0.14, 0.09]
$\text{falls}_{9} \rightarrow \text{r\_fi}_{9}$	0.29 [–0.09, 0.67]
$\text{falls}_{9} \rightarrow \text{r\_ucla}_{9}$	–0.03 [–0.09, 0.02]
$\text{falls}_{10} \rightarrow \text{r\_fi}_{10}$	0.04 [–0.14, 0.23]
$\text{falls}_{10} \rightarrow \text{r\_ucla}_{10}$	–0.09 [–0.17, –0.00]
$\text{falls}_{11} \rightarrow \text{r\_fi}_{11}$	0.07 [–0.03, 0.17]
$\text{falls}_{11} \rightarrow \text{r\_ucla}_{11}$	–0.00 [–0.03, 0.02]
$\text{falls}_{12} \rightarrow \text{r\_fi}_{12}$	0.12 [–0.03, 0.28]
$\text{falls}_{12} \rightarrow \text{r\_ucla}_{12}$	–0.02 [–0.07, 0.02]
$\text{falls}_{13} \rightarrow \text{r\_fi}_{13}$	0.15 [–0.01, 0.31]
$\text{falls}_{13} \rightarrow \text{r\_ucla}_{13}$	–0.08 [–0.19, 0.03]
$\text{falls}_{14} \rightarrow \text{r\_fi}_{14}$	0.05 [0.03, 0.07]
$\text{falls}_{14} \rightarrow \text{r\_ucla}_{14}$	0.07 [0.03, 0.11]

Note. We report standardized parameter estimates and 95.00 % CI intervals for all variables, except for the means of fixed/random effects; here, we report unstandardized estimates, as indicated by *. Model fit: $\chi^2(688) = 1035.99$ $p < .001$; robust TLI = 0.947; robust CFI = 0.953; SRMR = 0.070; robust RMSEA [90% CI] = 0.052 [0.044, 0.059].

Supplementary References

Curran, P. J., Howard, A. L., Bainter, S. A., Lane, S. T., & McGinley, J. S. (2014). The separation of between-person and within-person components of individual change over time: A latent curve model with structured residuals. *Journal of consulting and clinical psychology*, *82*(5), 879. https://doi.org/10.1037/a0035297

McNeish, D., & Matta, T. (2018). Differentiating between mixed-effects and latent-curve approaches to growth modeling. *Behavior research methods*, *50*, 1398–1414. https://doi.org/10.3758/s13428-017-0976-5

Stolz, E. (2024). *FRequent health Assessment In Later life (FRAIL70+) (SUF edition)*. https://doi.org/10.11587/DJNOHX
